# Genomic Evolution and Selective Pressure Analysis of a Novel Porcine Sapovirus in Shanghai, China

**DOI:** 10.3390/microorganisms12030569

**Published:** 2024-03-12

**Authors:** Jie Tao, Benqiang Li, Ying Shi, Jinghua Cheng, Pan Tang, Jiajie Jiao, Huili Liu

**Affiliations:** 1 Institute of Animal Husbandry and Veterinary Medicine, Shanghai Academy of Agricultural Sciences, Shanghai 201106, China; livia_taojie@126.com (J.T.); libenqiang2007@163.com (B.L.); shiyingsunny@126.com (Y.S.); zero5cheng@163.com (J.C.); tangpan8811@163.com (P.T.); jiaojiajie98@163.com (J.J.); 2 Shanghai Key Laboratory of Agricultural Genetic Breeding, Shanghai 201106, China; 3 Shanghai Engineering Research Center of Pig Breeding, Shanghai 201302, China

**Keywords:** metagenomic sequencing, porcine sapovirus, genomic evolution, recombination, selective pressure

## Abstract

Porcine sapovirus (PoSaV) is one of the most significant pathogens causing piglet diarrhea, and one with limited genetic characterization. In this study, the prevalence, infection pattern, and genetic evolution of porcine sapovirus were elucidated in detail. The positive rate of PoSaV was 10.1% (20/198), with dual, triple, and quadruple infections of 45%, 40%, and 5%, respectively. To further explore the viral composition in the PoSaV-positive diarrhea feces, metagenomic sequencing was carried out. The results confirmed that RNA viruses accounted for a higher proportion (55.47%), including the two primary viruses of PoSaV (21.78%) and porcine astrovirus (PAstV) (24.54%) in the tested diarrhea feces samples. Afterward, a full-length sequence of the PoSaV isolate was amplified and named SHCM/Mega2023, and also given the identifier of GenBank No. PP388958. Phylogenetic analysis identified the prevalent PoSaV strain SHCM/Mega2023 in the GIII genogroup, involving a recombinant event with MK962338 and KT922089, with the breakpoint at 2969–5132 nucleotides (nt). The time tree revealed that the GIII genogroup exhibits the widest divergence time span, indicating a high likelihood of viral recombination. Moreover, SHCM/Mega2023 had three nucleotide “RPL” insertions at the 151–153 nt site in the VP2 gene, compared to the other GIII strains. Further selective pressure calculations demonstrate that the whole genome of the SHCM/Mega2023 strain was under purifying selection (dN/dS < 1), with seven positively selected sites in the VP1 protein, which might be related to antigenicity. In conclusion, this study presents a novel genomic evolution of PoSaV, offering valuable insights into antigenicity and for vaccine research.

## 1. Introduction

Sapovirus (SaV), a member of the *Caliciviridae* family alongside Norovirus, Lagovirus, and Vesivirus, stands out as a leading cause of acute gastroenteritis in humans, especially among younger children [1]. SaVs, with a non-enveloped structure, possess a linear genome comprising polyadenylated, single-stranded, positive-sense RNA. The whole genome of SaV consists of about 7–8000 nucleotides (nt), excluding a 3′-end polyadenylated tail, with a small virus-encoded protein (VPg) at the 5′-end. In general, the SaV genome features two primary open reading frames (ORFs): ORF1 encodes the non-structural proteins NS1-NS2-NS3 (NTPase)-NS4-NS5 (VPg)-NS6 (protease)-NS7 (RNA-dependent RNA polymerase (RdRp)) and the capsid protein VP1; ORF2 encodes the minor capsid protein VP2 [2]. However, some specific genogroup strains have an additional ORF (ORF3) with an unknown function [3]. It has been confirmed that the capsid gene sequences of SaVs appear to have great genetic variability [4]. Genetically diverse, SaVs fall into 19 genogroups based on their VP1 sequences [5].

SaVs have been identified globally [6,7,8], and also in various species such as swine [9], mink [10], cows [11], dogs [12], and bats [13]. Porcine SaV (PoSaV) was first observed in fecal specimens from piglets in the USA, by researchers using electron microscopy, in 1980 [14]. An initial gastroenteritis outbreak in Chinese piglets caused by PoSaV was reported in Shanghai in 2008 [15]. In the present day, PoSaV has become one of the most common diarrhea pathogens in pigs. Despite its global presence, genomic characterization in China remains limited and its pathogenicity mechanism is still unknown. Some challenges exist for our ability to better understand sapovirus infections, including the inability to grow sapoviruses in cell cultures, which has hindered formal diagnosis and studies of immunity. Another challenge is that individuals with sapovirus infection are commonly co-infected with other enteric pathogens, complicating our ability to attribute the diarrhea episode to a single pathogen [16]. Nevertheless, there is still a need to monitor the epidemic dynamics and variation characteristics of PoSaV. Genetic diversification is a dynamic and complex process. The viral genome, in the different populations, may be under different selection pressures, purifying/positive or neutral [17]. Thus, it is important to assess the selection pressure of different micropopulations of viral genomes to understand the origin of genetic diversification.

In this study, 198 diarrhea feces of piglets were collected in Shanghai, China, for diarrhea pathogen surveillance. The results showed that co-infections were severe in the PoSaV-positive samples. Furthermore, metagenomics analysis was further carried out to explore the viral composition in these PoSaV-positive feces. It was revealed that PoSaV and porcine astrovirus (PAstV) were the two main pathogens in the feces. In addition, a full-length sequence of a novel recombinant GIII PoSaV was amplified, and its genetic divergence and selective pressure were analyzed comprehensively. This reminds us that attention should be paid not only to the porcine epidemic diarrhea virus (PEDV) but also to other pathogens, e.g., PoSaV and PAstV. Though they are also found in healthy pigs, co-infection may cause severe clinical diarrhea. 

## 2. Materials and Methods

### 2.1. Samples and Background Information

In 2023, 198 diarrhea feces of piglets were collected from different pig herds in Shanghai, China, and then subjected to the multiplex RT-PCR detection established in our laboratory. Then, 20 PoSaV-positive feces derived from three pig herds were divided into two groups: PCM1 and PCM2. The nine diarrhea feces derived from pig herd A were classified into PCM1. The six diarrhea feces derived from pig herd B and five feces from pig herd C were classified into PCM2 because of their proximity.

### 2.2. Metagenomic Sequencing and Data Analysis

Genomic DNA/RNA of PCM1 and PCM2 were extracted from the pretreatment samples through the use of a MagPure Viral DNA/RNA Mino LQ Kit (Maggigene, Guangzhou, China). The viral RNA underwent reverse transcription by SuperScript III reverse transcriptase (Invitrogen, Carlsbad, CA, USA). Subsequently, the SMARTer Ultra Low Input RNA kit (TAKARA, Shiga, Japan) was used to synthesize double-strand cDNA, and the quality of viral nucleic acids was assessed using a NanoDrop spectrophotometer (Thermo Fisher Scientific, Waltham, MA, USA) and 1.5% agarose electrophoresis. The Next^®^ UltraTM DNA Library Prep Kit for Illumina^®^ (New England Biolabs, MA, USA) was employed for generating sequencing libraries [18]. High-throughput sequencing occurred on an Illumina Novaseq 6000, with 150 bp paired-end reads being produced by the Magigene Company (Guangzhou, China).

The raw sequencing reads were processed to obtain clean data using Soapnuke (v2.0.5) for further analysis [19]. Quality-filtered reads were then de novo assembled to generate the metagenome. Different virus families were classified according to the annotation information in the NCBI taxonomy database.

### 2.3. Complete Genome Sequencing of PoSaV and Genomic Evolutionary Analysis

We extracted the total RNA using TRIzol reagent (TAKARA, Shiga, Japan), followed by cDNA synthesis using the downstream primer (5′-CGGTACGCGTAACCAGGGAAAGA-3′); a full-length sequence of PoSaV was amplified by means of RT-PCR. Three primer pairs were designed based on the reference sequence KT922089. The amplification conditions involved initial denaturation at 98 °C for 2 min, followed by 30 cycles of 98 °C for 20 s, 60 °C for 20 s, and 72 °C for 30 s, with an additional extension step at 72 °C for 5 min. The 5′ and 3′ end sequences were detected using a 5′-RACE kit and 3′-RACE kit (TAKARA, Japan) [20], respectively. Each fragment underwent cloning into a pEASY Blunt Zero Vector and sequencing by Sangon Biotech (Shanghai) Co., Ltd. (China). Furthermore, the full-length sequence was subsequently compared and spliced using Clustal and Lasergene 7.0 software.

To evaluate the genetic characteristics and relationships of the PoSaVs, ORF2 genes of the available PoSaVs retrieved from GenBank were analyzed through a neighbor-joining phylogenetic tree, using interactive Tree Of Life (iTOL) v3 software http://itol.embl.de/ (accessed on 15 January 2024) [21]. To analyze the antigenicity and immunogenicity of PoSaV, the amino-acid homology of VP2 proteins was compared using MegAlign software (Lasergene v7.1.0, DNASTAR, Inc., Madison, WI, USA). Furthermore, another phylogenetic tree and time tree were constructed based on RdRp sequences using the maximum-likelihood method within MEGA 11, with 1000 bootstrap replicates. A 50% cut-off value was applied to condense the tree. The RelTime with Dated Tips (RTDT) method estimated divergence times for all tree-branching points [22]. Fifty PoSaV strains were referred to and identified between 1998 and 2022 in the time tree.

### 2.4. Recombination and Selective Pressure Analysis

The viral recombination of the whole genome of PoSaV was carried out using the Recombination Detection Program (RDP) software, version 4 [22]. Furthermore, the amino-acid homology of the PoSaV ORF1 proteins was assessed using Lasergene v.7.1.0 and MEGA v.11 for selective pressure (SP) analysis. SP was measured by the ratio of the number of nonsynonymous substitutions per nonsynonymous (dN) to the number of synonymous substitutions per synonymous site (dS) using BUSTED method of the Datamonkey online software http://www.datamonkey.org/ (accessed on 20 January 2024). A dN/dS = 1 represents neutal evolution, and a dN/dS ration significantly greater than one provides evidence of positive selection, conversely is negative selection [23].

## 3. Results

### 3.1. Sapovirus Had High Viral Contents in the Diarrhea Feces of Piglets

**Figure 1:**
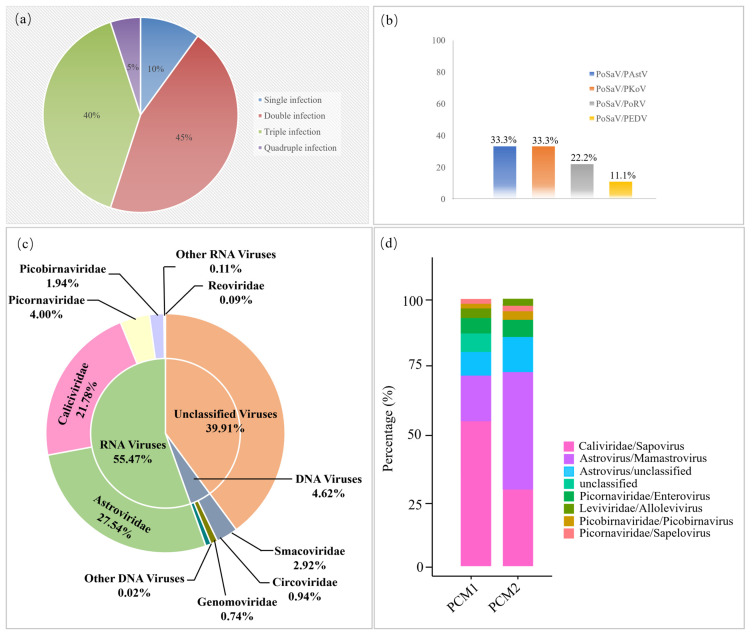
Viral composition of the diarrhea feces of piglets. (**a**) A total of 198 piglet diarrhea feces samples were detected by multiplex RT-PCR. The single infection and co-infection rates of PoSaV were analyzed. (**b**) Dual-infection patterns of the PoSaV-positive samples were further analyzed. (**c**) The 20 PoSaV-positive samples derived from three pig herds were divided into two groups (PCM1 and PCM2) for metagenomic sequencing. PCM1 contained the nine PoSaV-positive feces derived from pig herd A. PCM2 contained the six PoSaV-positive feces derived from pig herd B and the five PoSaV-positive feces from pig herd C. Then, the taxonomic distributions of the RNA virus, DNA virus, and unclassified viruses were calculated. (**d**) Finally, the RNA virus ratios in the PCM1 and PCM2 samples were determined.

In 2023, 198 diarrhea feces of piglets were collected and detected using a multiplex RT-PCR assay constructed in our laboratory. The results showed that the PoSaV positive rate reached 10.1% (20/198). Among the twenty PoSaV-positive samples, only two samples were infected with a single pathogen. Double-infection and triple-infection rates reached 45% and 40% (Figure 1a), respectively. Among the double infections, the PoSaV/PAstV, PoSaV/PoRV, PoSaV/PKoV, and PoSaV/PEDV co-infection rates were 33.3%, 33.3%, 22.2%, and 11.1% (Figure 1b), respectively. 

To further explore the viral composition characteristics of the 20 PoSaV-positive feces, two groups of PCM1 and PCM2 were sequenced via metagenomics. The results confirmed that the viral compositions of PCM1 and PCM2 were similar. The data of the two groups were analyzed together and they revealed that RNA viruses (55.47%) demonstrated a higher percentage than DNA viruses (4.62%), except for the unclassified viruses. RNA viruses identified included *Caliciviridae* (27.54%), *Astroviridae* (21.78%), *Picornaviridae* (4.00%), *Picobirnaviridae* (1.94%), *Reoviridae* (0.09%), and other RNA viruses (0.11%). DNA viruses identified mainly included *Smacoviridae* (2.92%), *Circoviridae* (0.94%), *Genomoviridae* (0.74%), and other DNA viruses (0.02%). Sapovirus emerged as the sole member of the family *Caliciviridae*. Analysis of the sapovirus ratios among all RNA viruses confirmed infection rates of 55.36% and 29.89% in PCM1 and PCM2, respectively (Figure 1b). 

### 3.2. Phylogenetic and Time Evolutionary Analysis

To understand the genomic evolution of sapovirus in piglet diarrhea, a full-length sequence of sapovirus was obtained through RT-PCR amplification and named SHCM/Mega2023. The full-length sequence of SHCM/Mega2023 (GenBank No. PP388958) contained 7338 nucleotides (nt), encoding the 2255 amino acid (aa) ORF1 and 174 aa ORF2. The ORF1 and ORF2 sequences had four nucleotides (ATGA) overlapped. SHCM/Mega2023 contained the conserved amino-acid motifs “GLPSG” and “YGDD” of calicivirus RdRp. The ORF1 protein of SHCM/Mega2023 had the highest homology with the PoSaV strain JJ259 (KT922089) and shared a 93.8% amino-acid identity. However, its ORF2 protein had the highest amino-acid identity with the PoSaV strain MO13472 (MK965905) (90.6%) and shared only an 83.6% amino-acid identity with JJ259. Compared with the capsid protein, JJ259 had a 9-aa insertion (GVTTTPKPQ), and MO13472 had a 7-aa insertion (GITPRPQ), while SHCM/Mega2023 had a 10-aa insertion (GITSRPLRPQ). 

**Figure 2:**
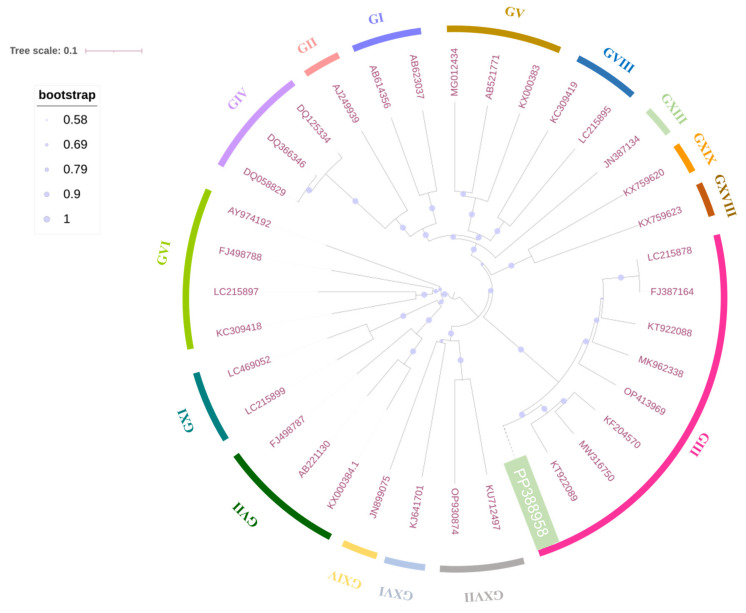
Phylogenetic analysis of the ORF2 genes of PoSaVs. A phylogenetic tree was constructed from the ORF2 genes of SHCM/Mega2023 and another 35 PoSaVs retrieved from GenBank, using the neighbor-joining method, and annotated using the interactive Tree Of Life (iTOL) software. The outside layer denotes the genogroups. The green box (SHCM/Mega2023) indicates the PoSaV sequenced in this study. The shaded dots indicate the bootstraps.

**Figure 3:**
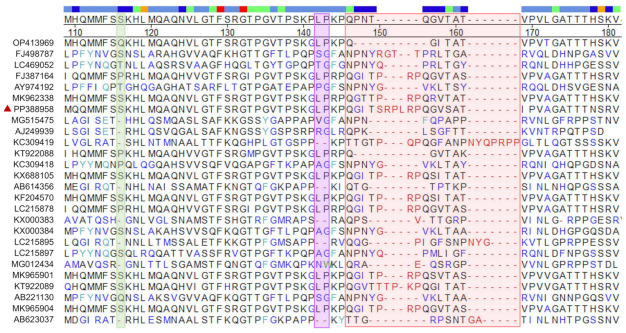
Amino-acid comparison of VP2 proteins of PoSaVs, using MegAlign software. The green, purple, and red rectangles are the three deletion sites. The labeled red triangle is the PoSaV isolate SHCM/Mega2023 sequenced in this study.

To explore its genetic characteristics, a phylogenetic tree involving SHCM/Mega2023 and corresponding GenBank strains based on the ORF2 genes was constructed. The results confirmed that SHCM/Mega2023 was classified into the GIII genotype, and it had the closest relationship with the 2016 USA-isolated JJ259 strain (KT922089) (Figure 2). The amino-acid comparison of the VP2 protein revealed that there were three deletion sites: 116 aa, 142–143 aa, and 146–169 aa (Figure 3). SHCM/Mega2023 (PP388958) had no deletions at the 116 aa site or the 142–143 aa site. Moreover, SHCM/Mega2023 (PP388958) had “RPL” inserted at the 151–153 aa site, while most PoSaV GIII strains had three amino acids detected at this site, which might exert potential effects on the immunogenicity of PoSaVs.

**Figure 4:**
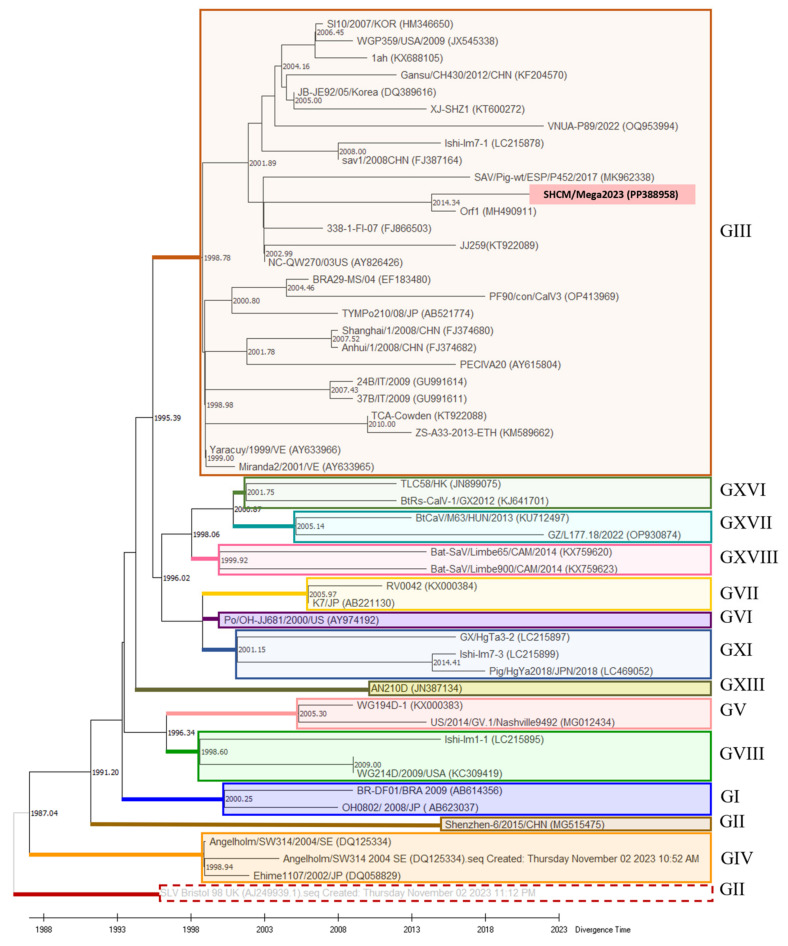
Evolutionary time-tree generated based on the RdRp genes of PoSaVs. The time tree was calculated in MEGA X, and the divergence time was inferred via the RelTime with Dated Tips (RTDT) method. The GII genogroup strain SLV Bristol 98UK (AJ249939) (grey color) was designated as an outgroup taxon, with all sequences using the year of sampling as the tip date for calibration constraints. The divergence time of each branch is marked. Different PoSaV genogroups are circled in differently colored dotted boxes.

Subsequently, by employing the RTDT method based on the RdRp genes, a time tree was calculated to examine sapovirus divergence times (Figure 4). The GII genotype showed the earliest origin among the eighteen genotypes, dating back to the 1980s. GIV emerged in 1987. GXVII, GVII, and GV shared a common divergence time in 2005. GVIII distinguished itself between 1998 and 2009, while GXI emerged between 2001 and 2014. Notably, the GIII genotype exhibited the most extended span of the divergence times, ranging from 1998 to 2014. SHCM/Mega2023, along with Orf1 (MH490911), diverged around 2014. 

### 3.3. Recombinant Analysis

**Figure 5:**
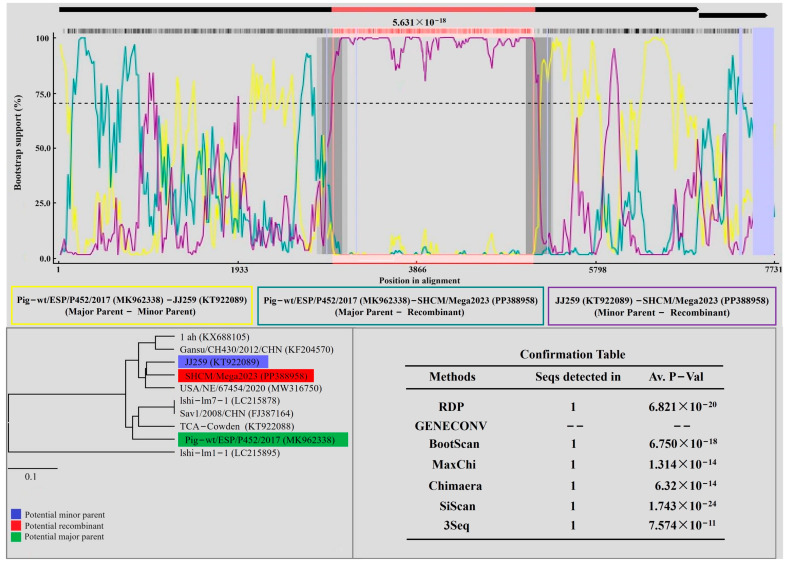
The recombination analysis of the complete SHCM/Meg2023 genome. The results were described using the RDP method, a finding which was supported by ≥6 programs to further characterize the potential recombination events. The pink box indicates the regions for the recombinant and its putative parents. The Y-axis represents the pairwise identity between the recombinant and its putative parents. The X-axis represents the position when aligned with a 30 nt sliding window. The comparisons of the recombinant–major parent, recombinant–minor parent, and major–minor parent are indicated by cyan, purple, and yellow lines, respectively.

In the complete genome recombination analysis, we utilized the RDP5 software to examine SHCM/Meg2023 and 33 reference strains. The results indicated that SHCM/Meg2023 potentially resulted from recombination between MK962338 (major parent) and KT922089 (minor parent), a finding supported by six detection algorithms (RDP 6.821 × 10^−20^, BootScan 6.750 × 10^−18^, MaxChi 1.314 × 10^−14^, Chimaera 6.32 × 10^−14^, SiScan 1.743 × 10^−24^, and 3Seq 6.574 × 10^−11^) (Figure 5). SAV/Pig-wt/ESP/P452/2017 (MK962338) and JJ259 (KT922089) are GIII PoSaV isolates from Spain (2017) and the USA (2016), respectively. Further analysis identified position 2969–5132 in the SHCM/Meg2023 genome as the predicted recombinant fragment, with the two breakpoints located within ORF1 (Figure 4).

### 3.4. Evolutionary Selective Pressures on the ORF1 Codons

**Figure 6:**
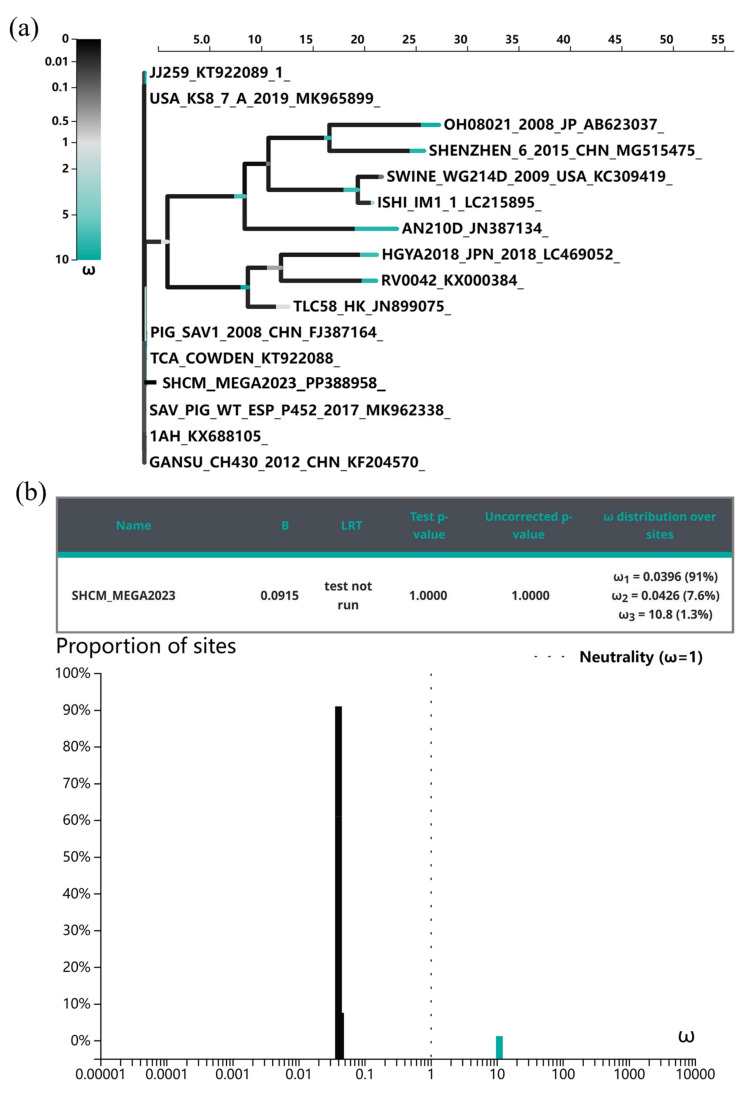
Selective pressure analysis of the ORF1 protein of PoSaVs. The genetic evolution of the ORF1 protein was first analyzed using Lasergene v.7.1 and MEGA v.11. Selection pressure was calculated using BUSTED method of the Datamonkey online software (http://www.datamonkey.org/, accessed on 15 January 2024) (**a**). Further the ω (dN/dS) distribution over sites were calculated (**b**). dN/dS > 1 represents positive selection, and vice versa is considered as purification selection.

To further evaluate the extent of selective pressure on the ORF1 gene, the standardized differences in dN/dS were calculated for each position. Scores > 0 suggest heightened diversifying selection, highlighting the propensity of ORF1 protein regions to diversify. Purifying selection (dN/dS < 1) was indicated by BUSTED models for SHCM/Mega2023 (Figure 6a). In the ORF1 protein, seven amino acids (2 aa, 110 aa, 529 aa, 620 aa, 1668 aa, 1977 aa, and 2232 aa) were subjected to diversifying positive selection (ω3 = 10.8), while 1314 amino acids underwent purifying selection (ω1 = 0.0396) (Figure 6b). Three positively selected sites (1668 aa, 1977 aa, and 2232 aa) were situated within ORF1 at capsid protein VP1.

## 4. Discussion

Pigs in all growth stages can contract PoSaVs, with a higher infection rate observed in post-weaning pigs [24,25]. No significant differences in SaV prevalence were found between age-matched groups of pigs, with and without diarrhea, in the field. However, PoSaVs frequently co-infect with other enteric pathogens, such as porcine epidemic diarrhea virus, porcine astrovirus, porcine kobuvirus, porcine delta coronavirus, etc. [26]. In this study, co-infection of PoSaV/PAstV and PoSaV/PoRV showed the highest rates in the PoSaV-positive feces samples, though the PoSaV-positive rate was only 10.1%. In previous research, we confirmed that both bovine viral diarrhea virus (BVDV) and porcine sapelovirus (PSV) could enhance PEDV pathogenicity [27]. Moreover, SaVs cause severe acute gastroenteritis in humans. Thus, we should pay attention to the co-infection of PoSaV and other diarrhea pathogens, which may also have a synergistic effect on the clinical pathogenesis of diarrhea disease.

To date, SaV has been categorized into 19 genogroups and 52 subtypes based on VP1 gene sequences, revealing four genogroups (GI, GII, GIV, and GV) and eight genogroups (GIII and GV-GXI) in humans and pigs, respectively [28]. GIII predominantly circulates in swine herds worldwide [21], which is consistent with our study’s findings. Through phylogenetic analysis, we identified the prevalent PoSaVs within the GIII genogroup. Given sapovirus’s current division into 19 genogroups, our study pioneered the calculation and comparison of their evolutionary rates. The results affirmed GIII’s having the widest span of divergence among the genogroups, spanning from 1998 to 2014. The divergence time of the SHCM/Mega2023 isolate was 2014.34, while another PoSaV strain identified in Shanghai (FJ374680) underwent divergence at the time point of 2007.52. Although the PoSaVs in the same area have the same genotype, their genomes are constantly evolving. The data confirmed that GIII exhibits the highest rates of transmission and variation. Genetic diversification also appeared in the VP2 proteins; most PoSaV GIII strains have three amino-acid deletions at the 151–153 aa site, while SHCM/Mega2023 had an “RPL” insertion at this site. Studies have reported that different deletion patterns may exert different effects on the immunogenicity of PoSaV [29]. Therefore, the PoSaV GIII isolates reported in this study have novel genetic and antigenic characteristics. However, the relationship between the novel recombinant event and pathogenesis remains unknown and requires further exploration. 

Recombination, acknowledged as an evolutionary force, can yield new viruses, each with a distinct pathogenesis and virulence [30]. Intra- and inter-genogroup recombinant strains have been reported [9,31,32]. Herein, we present the novel PoSaV isolate SHCM/Mega2023, characterized by recombination with MK962338 (major parent) and KT922089 (minor parent), with a breakpoint identified at 2969–5132 nt in the RdRp-capsid junction region. The results suggested that intra-genogroup recombination may occur between field GIII strains, a premise which was consistent with most of the recombination events [15,33,34,35,36]. Available sequences showed that Yunnan PoSaV-GIII strain YNAN might be the only recombinant with breakpoints located in the VP1 region, which is important for immunogenicity [34]. And it also had evolved via an intra-genogroup recombination event. This revealed that PoSaV-GIII is still the predominant genotype prevalent in pig herds of Shanghai; presently, the PoSaV-GIII strains are mostly recombinant between intra-groups. It is well-known that virus recombination analysis can state explicitly whether the recombination events occur on an intra-genogroup basis or an inter-genogroup basis, which elucidate the variations of the epidemic strains. This can not only provide critical information for vaccine development, but also evaluate whether the existing vaccines are compatible with the epidemic strains. Additionally, detailed genomic homology analysis should also be performed, especially for the amino-acid sites which have an influence on the immunogenicity. Although both the SHCM/Mega2023 and p2 strains were reported in Shanghai and exhibited similar recombination events, the complete-genome homology of the SHCM/Mega2023 and p2 strains was only 84.8%, which confirmed the great diversity between them. Moreover, SHCM/Mega2023 had distinct insertion in the VP2 region, which might have effects on the immunogenicity of PoSaV, and needs to be further explored. 

Investigation into the selective pressures acting on a gene is crucial for comprehending its evolutionary dynamics and may unveil potential drug-targeting regions, as amino-acid substitutions at negatively selected sites are likely intolerable [37,38]. Positively selected sites aid in identifying epitope-contacting residues [39]. Meanwhile, the presence of positive selection signatures in the genome is a characteristic indication of adaptation, revealing ongoing, recent, or ancient responses to environmental changes throughout a population’s evolution [40]. In SaV, the polyprotein encoded by open reading frame 1 (ORF1) undergoes cleavage to form various viral proteins, including 2C-like nucleoside triphosphatase (NTPase), 3C-like protease (Pro), virus-genome linked protein (VPg), 3D-like RNA-dependent RNA polymerase (Pol), and the major capsid protein (VP1) [41]. In this study, the selective pressure force of the ORF1 protein of PoSaVs was calculated, initially revealing seven positively selected sites in the ORF1 protein of SHCM/Mega2023. Notably, three positively selected sites (1668 aa, 1977 aa, and 2232 aa) within the capsid protein VP1 of ORF1 are potentially associated with antigenicity. VP1, the main structural protein of PoSaV, safeguards the viral nucleic acid and features multiple immunogenic epitopes that elicit both humoral and cellular immunity. 

In conclusion, we found that co-infection of PoSaV/PAstV and other diarrhea pathogens was severe, though the PoSaV-positive rate was only 10.1%. In addition, we carried out a detailed evolutionary analysis of the prevalent PoSaV isolate SHCM/Mega2023 (PP388958), derived from the diarrhea feces of piglets in Shanghai. The data in this study highlight the genetic diversity of PoSaV, and attention should be paid to the evolution and co-infection of PoSaV.

## Data Availability

Data is contained within the article.
